# Therapy effect of cochleural alternating acoustic beam therapy versus traditional sound therapy for managing chronic idiopathic tinnitus patients

**DOI:** 10.1038/s41598-024-55866-0

**Published:** 2024-03-11

**Authors:** Chunli Liu, Jie Zhang, Zhiwei Qi, Wenhui Yue, Yujie Yuan, Tao Jiang, Shenglin Zhang, Shujun Zhang

**Affiliations:** 1 Department of Otolaryngology, The Affiliated Hospital of Chengde Medical College, Chengde, 067000 Hebei People’s Republic of China; 2 The Labs of Micro-DSP Technology LTD, Fl 10, Tower C, 136 Bin Jiang Dong Lu, Chengdu, 610021 People’s Republic of China; 3 Department of Oncology, The Affiliated Hospital of Chengde Medical College, Chengde, 067000 Hebei People’s Republic of China

**Keywords:** Cochleural alternating acoustic beam therapy, Sound therapy, Chronic idiopathic tinnitus, Randomized controlled clinical trial, Diseases, Health care, Medical research

## Abstract

Idiopathic tinnitus is a common and complex disorder with no established cure. The CAABT (Cochleural Alternating Acoustic Beam Therapy CAABT), is a personalized sound therapy designed to target specific tinnitus frequencies and effectively intervene in tinnitus according to clinical tinnitus assessment. This study aimed to compare the effectiveness of the CAABT and Traditional Sound Therapy (TST) in managing chronic idiopathic tinnitus. This was a randomized, double-blind, parallel-group, single-center prospective study. Sixty adult patients with tinnitus were recruited and randomly assigned to the CAABT or TST group in a 1:1 ratio using a computer-generated randomization. The treatment lasted for 12 weeks, and participants underwent assessments using the tinnitus handicap inventory (THI), visual analog scale (VAS), tinnitus loudness measurements, and resting-state functional magnetic resonance imaging (rs-fMRI). Both groups showed significant reductions in THI scores, VAS scores, and tinnitus loudness after treatment. However, CAABT showed superiority to TST in THI Functional (*p* = 0.018), THI Emotional (*p* = 0.015), THI Catastrophic (*p* = 0.022), THI total score (*p* = 0.005) as well as VAS score (*p* = 0.022). More interesting, CAABT showed superiority to TST in the changes of THI scores, and VAS scores from baseline. The rs-fMRI results showed significant changes in the precuneus before and after treatment in both groups. Moreover, the CAABT group showed more changes in brain regions compared to the TST. No side effects were observed. These findings suggest that CAABT may be a promising treatment option for chronic idiopathic tinnitus, providing significant improvements in tinnitus-related symptoms and brain activity.

Trial registration: ClinicalTrials.gov:NCT02774122.

## Introduction

Idiopathic tinnitus is a heterogeneous disorder that affects millions of individuals worldwide across all age groups; its prevalence in adults can reach 15%^1^. Recent research has sought to differentiate between tinnitus and tinnitus disorders. Because of the close associations between tinnitus and psychological disorders (e.g., anxiety, depression, and insomnia), tinnitus disorders are defined as functional impairment and behavioral changes resulting from psychological disorders (e.g., anxiety, depression, and insomnia) when tinnitus^2^. For example, negative emotions not only act as a susceptibility factor but also ensue as a consequence of tinnitus, thereby intensifying the severity of the condition and hindering patients' adaptation to the persistent sound. Hearing loss linked to cognitive decline and poorer cognitive performance is caused by severe' tinnitus. Additionally, autonomic dysfunction is also known as a risk factor for tinnitus. Excessive stimulation of the sympathetic nervous system heightens mental and physical tension, potentially lowering tolerance to tinnitus and impeding habituation. Recent studies have investigated the mechanism of tinnitus, to develop effective management strategies; tinnitus was presumed to originate in the cochlea. A reduction in cochlea nerve afferent input triggers compensatory changes in neurotransmission within auditory and non-auditory structures, leading to neural hyperexcitability. These persistent changes in neural hyperexcitability contribute to the formation of tinnitus networks characterized by altered functional connectivity, resulting in the development and perception of chronic tinnitus^2^. However, perceptions of tinnitus and external sounds involve distinct and independent pathways. The bottom-up pathway is responsible for processing external sounds, whereas the Top-down pathway is involved in the perception of tinnitus. Consequently, the mechanism underlying chronic tinnitus focuses primarily on functional connections between higher-level auditory and non-auditory centers^3–5^.

Despite the strong preference for drug therapy in tinnitus management in China, no specific drug or cure has been established^6^. However, certain interventions enhance the quality of life and reduce tinnitus-related psychological distress by addressing the impact of tinnitus sensations. Cognitive behavioral therapy, recommended in the American tinnitus guidelines, has shown positive therapeutic effects in terms of improving quality of life and reducing the adverse effects of tinnitus. However, its effectiveness may be limited in cases of severe or prolonged tinnitus^7^. Sound therapy, assessed through subjective evaluations, has demonstrated effectiveness in tinnitus management. The efficacy of sound therapy improves with the duration of daily treatment; it can be effective as a standalone method without additional counseling^8,9^. Vernon^10^ demonstrated that tinnitus masking, a standard monotone therapy, involves using external noise to distract the patient's attention and reduce the contrast between the tinnitus signal and background activity within the auditory system. Advancements in portable devices, such as smartphones, have led to the development of cochleural alternating acoustic beam therapy (CAABT)^11^; this method uses various sound therapy algorithms to modify the sound in all dimensions, adapting to the patient’s needs over time. It is a sound therapy that focuses on specific tinnitus frequencies and relies on clinical tinnitus assessment methods for intervention. We aimed to provide alternative options for patients seeking personalized intervention. Furthermore, based on clinical assessment methods, sound therapy adjusted according to specific tinnitus frequencies offers personalized interventions. Compared with other sound therapies, CAABT could tailor acoustic stimuli to the unique characteristics of each patient's tinnitus perception. Tailored sound therapy, in general, aims to provide auditory stimuli customized to an individual's specific tinnitus features. CAABT goes a step further by incorporating adaptive beamforming technology. It dynamically adjusts the acoustic beam to match the perceived characteristics of the tinnitus for each patient, providing a more individualized and potentially effective treatment. The "alternating" aspect in the context of CAABT may refer to the dynamic adjustments or variations made to the acoustic beam, possibly involving changes in frequency, intensity, or spatial characteristics. These alterations aim to optimize the therapeutic effects of acoustic stimulation for better outcomes in managing tinnitus. Therefore, in this randomized controlled trial, we compared the effectiveness of CAABT and traditional sound therapy (TST).

Advancements in neuroimaging techniques, particularly functional magnetic resonance imaging (fMRI), have provided valuable insights into tinnitus-related changes in brain structure and function. Resting-state fMRI (rs-fMRI), based on blood-oxygenation-level-dependent (BOLD) signals, assesses abnormal spontaneous activity in specific brain regions of individuals with tinnitus during the resting state. It is well-recognized that rs-fMRI is a highly effective, non-invasive method for investigating the neural pathways associated with tinnitus. It can also be utilized for therapeutic efficacy assessments^7^. This non-invasive technique provides objective and detailed data regarding spontaneous neural activity, functional connectivity, and altered brain function. Notably, rs-fMRI can investigate neural pathways associated with tinnitus and evaluate treatment efficacy^12^. Zang et al.^13^ introduced the use of regional homogeneity (ReHo) analysis in fMRI. ReHo analysis measures consistency in differences between a specific voxel and its neighboring voxels during the resting state. The synchronization of local neural activities can be investigated by comparing changes in ReHo values in a particular brain region among healthy individuals, which reveals the involvement of these brain areas in neural processing^14^. The fMRI results revealed distinct patterns of activation within key auditory and limbic regions, shedding light on the neural correlates of this enigmatic condition. To elucidate the neurobiological underpinnings of tinnitus, our investigation employed fMRI to probe the changes in neural activity associated with tinnitus perception after both interventions.

## Methods

This two-group, double-blind, randomized controlled trial compared the effectiveness of CAABT and TST in the management of adult patients with tinnitus at the Department of Otolaryngology-Head and Neck Surgery, Beijing Friendship Hospital, Capital Medical University, using a 12-week follow-up period after treatment. The trial was registered at ClinicalTrials.gov (NCT02774122) in Aug. 2016. The Ethics Committee of Beijing Friendship Hospital approved the study protocol (approval no. 2016P2-012). The study was performed in accordance with the ethical standards as laid down in the 1964 Declaration of Helsinki and its later amendments or comparable ethical standards.

### Inclusion and exclusion criteria

Inclusion criteria:

1. Participants aged 18–80 years with unilateral chronic tinnitus lasting for at least 6 months;
2. All subjects had the dominant tinnitus frequency between 125 and 8000 Hz;
3. Hearing loss did not exceed moderate deafness and communicated normally;
4. Persistent tinnitus;
5. Patients with primary or idiopathic tinnitus.

We excluded participants with secondary tinnitus resulting from external, middle, or inner ear diseases, auditory nerve disease, or non-auditory system dysfunction; hyperacusis; contraindications for magnetic resonance imaging; The ears with tinnitus had moderately severe or severe hearing loss; serious mental illness; incomplete adherence to the prescribed treatment; receipt of alternative sound therapies; or inability to cooperate during the audiological examination and tinnitus detection. Additionally, patients with acoustic neuroma were excluded after acoustic emissions (DPOAE) and whole-brain morphological examinations using a 3-Tesla magnetic resonance imaging device (General Electric; Milwaukee, WI, USA).

### Participant recruitment

Volunteers and unilateral tinnitus patients who visited Beijing Friendship Hospital were recruited for unilateral tinnitus sound therapy through newspaper advertisements, online platforms, and email. We collected data on all outcomes at 12-week follow-up. After routine registration, all patients underwent physical examinations performed by otolaryngologists, as well as audiological and psychoacoustic tinnitus evaluations. Eligible patients completed the case record form, tinnitus handicap inventory (THI), and visual analogue scale (VAS). Informed consent was obtained from all study participants.

### Randomization of CAABT and sound therapy groups

Group randomization was conducted using a validated computer-generated randomization table. Participants were informed that they would be randomly assigned to receive a harmless sound therapy for tinnitus treatment. Subsequently, an independent researcher not involved in the clinical trial assigned participants to treatment groups at a 1:1 ratio based on the randomization table. Participants, investigators (clinicians and audiologists), and statisticians were unaware of the treatment group to which each patient had been assigned (cochleural alternating acoustic beam therapy or sound therapy). To ensure allocation concealment, the sound therapy devices were identical in terms of packaging, labeling, appearance, and wearing schedule.

### Interventions (CAABT or TST) and tinnitus consultation

CAABT, an innovative synthetic sound therapy, transmits two sound components to patients using specialized equipment. The first component comprises natural sounds such as ocean or water sources, allowing patients to select their preferred background sounds. This harmonious natural sound background helps patients adapt to the treatment, desensitizes their reaction to tinnitus, reorganizes the central system to cope with tinnitus, and reduces hypersensitivity. Similar to acoustic coordinated reset neuromodulation(ACRN), the second component focuses on tinnitus frequency (TF), as identified through acoustic tinnitus evaluations. Sounds centered around TF generate four groups of sounds: F1, TF, F2, and ST (F1 + TF + F2) where F1 represents TF (1–10%) Hz, F2 represents TF (1 + 10%) Hz, and ST represents simultaneous sound stimulation of F1, TF, and F2. The sound sequence is arranged as F1, TF, F2, and ST (F1 + TF + F2), and this sequence is repeated. We disrupted auditory memory associated with tinnitus, attenuated unpleasant experiences, and induced brain plasticity by alternating the TF based on stimulation^15^. Each sound lasts 150 ms; intervals between stimulus sounds are set to 100, 100, 500, and 500 ms for D1, D2, D3, and D4, respectively (Supplementary Fig. 1). The intensities of the stimulus sound and background sound were determined based on the minimum masking level (MML); the stimulus sound was set to MML—5 dB and the background sound was set to MML—10 dB (Supplementary Fig. 2). Patients can effortlessly listen to these sounds by wearing sound emitters and activating the switch.

TST, a conventional monophonic sound therapy, typically involves the use of narrow-band noise that matches the TF. The intensity of the masking sound exceeds the MML of tinnitus by 5 dB. TST remains a commonly used therapy for tinnitus because of its simplicity and safety. The therapeutic mechanism involves introducing a background sound that weakens the ability of the nervous system to perceive the tinnitus sound, thereby reducing its impact. Additionally, external sounds distract the patient's attention away from the tinnitus, resulting in reduced perception and distress. However, sound therapy has some limitations in clinical practice. The monotonous nature of the narrow-band noise used in masking may render it unappealing to some patients, who find it tedious to listen to masking sounds that closely resemble their tinnitus frequency. Furthermore, issues such as separation-type masking curves, or challenges in achieving effective masking can limit its effectiveness. CAABT introduces a unique auditory experience by incorporating two integral components: synthesized background sound and stimulus sound, diverging from the conventional use of narrowband noise. The fluctuating nature of the background sound, characterized by dynamic variations, ensures that recipients do not experience monotony or tedium. Notably, CAABT's auditory composition avoids the partial masking of tinnitus sounds, thereby circumventing constraints associated with specific masking curve types.

Tinnitus counseling for patients involves explaining the causes, potential harms, and effective management strategies; it also involves emphasizing the significance of maintaining the audibility of the tinnitus side of the ear as well as the use of sound generators and an understanding of the specific mechanisms by which tinnitus can be habituated. Moreover, it is important to share the experiences of individuals with tinnitus who have benefited from sound therapy.

### rs-fMRI

fMRI was conducted using BOLD signals, a common approach. All participants underwent whole-brain morphological examinations using a 3-Tesla magnetic resonance imaging device (General Electric; Milwaukee, WI, USA) in the Department of Radiology at Beijing Friendship Hospital, Capital Medical University. Scans were conducted with a 16-channel phase control head coil while the participants were in a supine position. During scans, participants were given rubber earplugs and earphones with outer covers to minimize external auditory stimuli. Participants were instructed to keep their heads still, close their eyes, and avoid thinking of anything in particular. The scanning parameters included three-dimensional fast spoiled gradient echo sequence imaging with a repetition time of 8.8 ms, echo time of 3.5 ms, field of view of 240 mm × 240 mm, slice thickness of 1.0 mm, slice distance of 0 mm, matrix size of 256 × 256, flip angle of 15°, and excitation number of 1.0. In total, 196 continuous sagittal plane images were obtained.60 normal individuals with the same sex, age, and handedness as all tinnitus patients were enrolled as the reference group. For tinnitus patients and 60 eligible normal participants, rs-fMRI was performed using a gradient-echo echo-planar imaging sequence with a repetition time of 2000 ms, echo time of 35 ms, layer thickness of 6 mm, field of view of 240 mm × 240 mm, layer spacing of 1 mm, matrix size of 64 × 64, flip angle of 90°, and excitation number of 1.0. In total, 200 time points were scanned, each lasting 2 s. The scan covered 28 layers from the base of the skull to the top of the head, with the scanning baseline parallel to the joint line of the front and back. rs-fMRI scans were performed free of charge; the costs of other tests were covered by medical insurance.

### Trial procedure

The experiment was conducted following a previously published randomized controlled trial protocol^16^. All participants underwent audiological and tinnitus-related examinations. Eligible participants completed the case record form and underwent rs-fMRI. Next, they were randomly assigned to the CAABT group or the TST group for 3 months. Before treatment, qualified audiologists instructed the participants to wear and adjust their sound therapy devices. The loudness of the therapy device was adjusted to the most comfortable level for each participant. Participants were advised to wear the sound therapy device for 15 min three times per day while maintaining a relaxed state without focusing on the sound signals. They were encouraged to engage in activities such as meditation, reading magazines, or internet browsing. The device volume was adjusted to a level that allowed the sound to be audible and blend with each participant’s tinnitus, rather than completely masking it. During treatment, participants were monitored each day through various instant messaging applications on their mobile phones. They received reminders and inquiries about device usage, such as "Did you wear the sound therapy device today?" and "Please continue using the therapy device according to the instructions for the entire 12-week treatment course." Participants were also encouraged to contact the researchers with any queries or concerns. Participants could withdraw from the trial at any time if they experienced discomfort or decided not to continue. The case record form collected basic personal information and details such as tinnitus duration, characteristics, triggers, and treatment history; affected side; handedness (left or right); and the impacts of tinnitus on thoughts, emotions, hearing, sleep, and attention.

### Outcome measures

The THI^17^, VAS, and tinnitus loudness were used as the main outcome indicators. Tinnitus impact on patients is assessed using THI, a commonly used clinical test, on three dimensions: functional, emotional, and catastrophic. It has 25 questions with a 3-point rating system (0 for no, 2 for occasionally, and 4 for yes). The total score is determined by summing the scores from all three dimensions, where higher scores indicate greater severity of tinnitus-related disorder^17,18^. Tinnitus severity is categorized into five grades (1 to 5), such that a higher grade indicates a higher score and a more severe condition. The maximum score achievable on the THI is 100 points. The validated Chinese version of the THI has demonstrated good reliability and validity, with a validity coefficient of 0.90^19^.

### VAS and tinnitus loudness

The VAS allows patients to subjectively assess the loudness of their tinnitus and its impact on their well-being using a rating scale, which ranges from 0 to 10^20^; higher scores indicate louder tinnitus sounds and greater impact. A score of 0 represents no tinnitus sound and no impact, whereas a score of 10 represents an infinitely loud tinnitus sound causing a catastrophic impact. Evaluation scores before and after treatment were compared to assess changes in tinnitus loudness.

Psychoacoustic measurements of tinnitus were conducted using the TiniTest system (www.micro-dsp.com), which includes comprehensive assessments, such as tinnitus loudness, pitch and loudness matching, octave confusion test, MML, and residual inhibition test. These tests were administered with precise intensity level and frequency to accurately identify specific therapy requirements. All hearing and tinnitus tests were performed in a soundproof room that complied with relevant standards (room noise ≤ 25 dB).

Our secondary outcome measure was the rs-fMRI.

### Feldmann's masking curves

Feldmann's masking curves were assessed through a test conducted at frequencies equivalent to those examined in standard tonal audiometry. This involved the use of narrow-band noises or pure tones (excluding cases where narrow-band noises failed to mask tinnitus). The test proceeded in 5 dB increments (lasting 1–2 s per stimulation) starting from hearing thresholds, and continuing until the participant indicated they could no longer perceive their tinnitus. The resulting curves, derived from the spatial relationship between hearing thresholds (Supplementary Table 1) and tinnitus masking, were categorized into five types according to Feldmann's classification: (1) Convergent; (2) overlapping; (3) Separated; (4) spaced; (5) Antagonistic.

### Statistical analysis

The sample size was calculated based on the THI score^21^; alpha and beta levels were 0.05 and 0.2 (two-sided), respectively, and the expected loss to follow-up rate was 20%. Based on the estimation that 58 participants were needed (29 per group), we enrolled 60 participants (30 per group). Statistical analyses were performed using SPSS 19.0 (IBM Corp., Armonk, NY, USA) following the consolidated standards guidelines^22^. Data was reported as the mean ± SD of triplicates. Categorical variables are represented as percentages and numbers. Comparative analyses within and between the CAABT group and TST group were conducted using independent samples t-tests. Paired samples t-tests were used for within-group comparisons; the chi-squared test was used for data with homogenous variance, whereas the Wilcoxon rank-sum test was used for data with heterogeneous variance. *P*-values < 0.05 were considered statistically significant.

Statistical analysis of fMRI data involved using the rs-fMRI data analysis toolkit (REST software; http://www.restfmri.net) to conduct ReHo analysis. The preprocessing steps included the removal of the first 10 volumes, temporal alignment, motion correction, spatial normalization, and resampling to a voxel size of 3 mm × 3 mm × 3 mm. After calculation, the ReHo results were spatially smoothed using a Gaussian filter with a maximum full width of 6 mm. The REST software automatically displayed the regional neural activity characteristics of tinnitus patients; the analysis revealed specific local neural activity characteristics within tinnitus patients. Statistical comparisons between groups were performed using analysis of covariance; differences in local neural activity between the groups before and after treatment were analyzed using paired-sample t-tests with false discovery rate correction. The significance threshold was set to *P* < 0.05. The results were presented using REST software.

## Results

### Baseline characteristics

**Figure 1:**
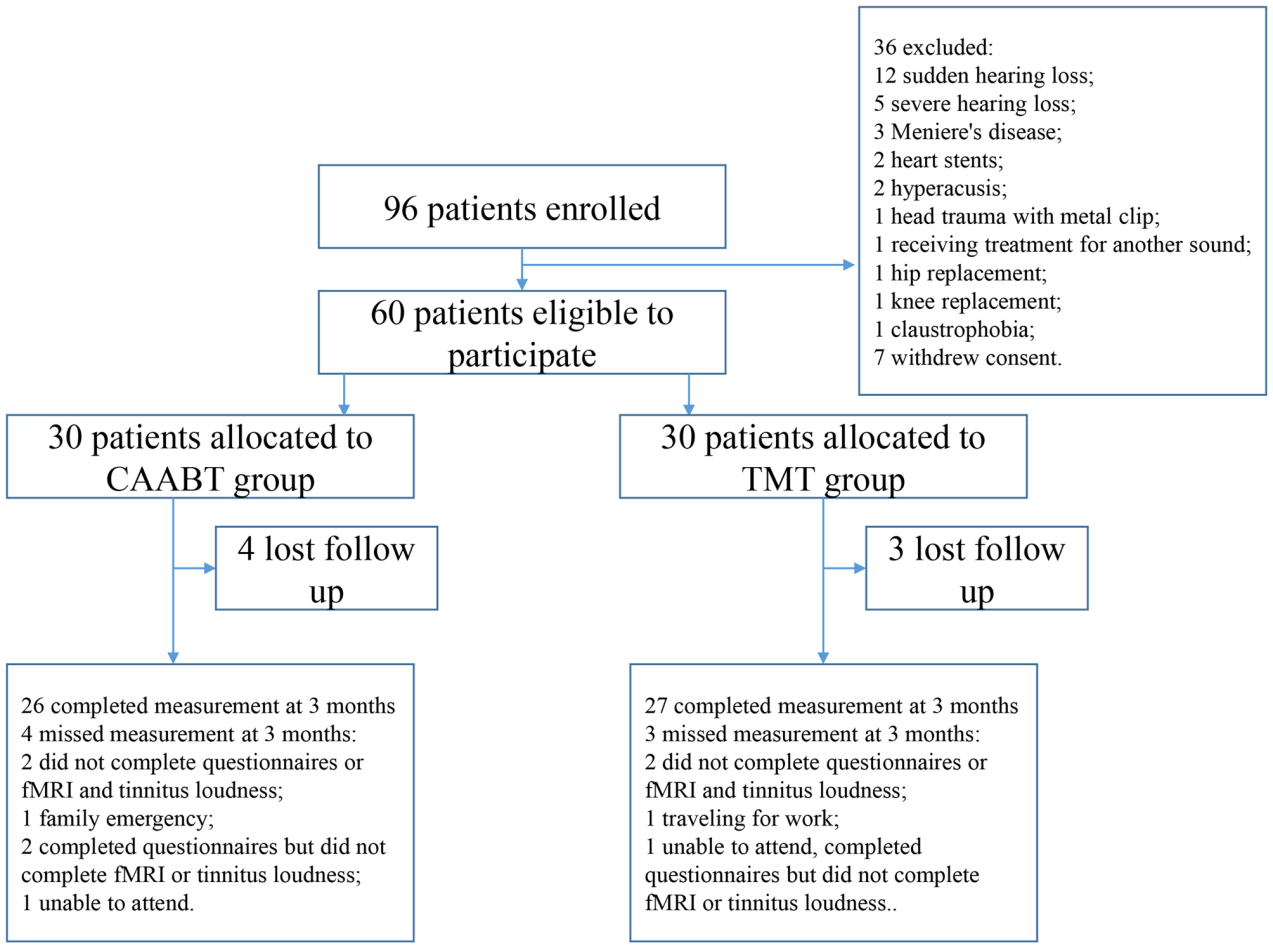
Flow diagram of the study protocol.

The study was conducted at the Department of Otorhinolaryngology-Head and Neck, Beijing Friendship Hospital, between March 2016 and July 2020. In total, 96 participants with unilateral persistent tinnitus were recruited. Of these, only 60 participants met the criteria for inclusion in the trial. In total, 60 participants were randomly assigned to the TST group or CAABT group (n = 30 per group). After excluding 4 CAABT patients and 3 TST patients from the group, 26CAABT patients and 27 TST patients were analyzed. Figure 1 illustrates the trial protocol, including participant withdrawal and non-response. Loss to follow-up did not significantly differ between the two groups (*P* > 0.6). Baseline characteristics of relevant covariates were assessed using completed questionnaires (THI, VAS) from both groups (n = 28 per group), except for patients who reported moderate tinnitus severity on the THI scale (Table 1). The distribution of patients between the CAABT and TST groups was well-balanced. The questionnaire responses indicated that all patients were engaged in very light physical activity (e.g., typing, driving, playing cards, and cooking) or light physical activity (e.g., walking on a flat surface, cleaning, and babysitting). Of the 56 participants, 36 (64.29%) had previously received tinnitus treatment. Furthermore, 20 participants (35.72%) reported severe and catastrophic tinnitus (total THI score ≥ 58). Additionally, 51 participants (91.07%) had pure tinnitus, 53 participants (94.64%) exhibited converging or overlapping Feldmann curves, and 44 participants (78.57%) showed complete positivity in the residual inhibition test.

**Table 1:** Baseline characteristics.

Characteristics and clinical outcomes	Mean (SD) (%)	CAABT group	TST group	X^2^/t	p
Age (years)	46.53 (13.22)	48.25 (14.23)	44.5 (12.21)	1.058	0.295
Ex (%)				0.664	0.415
Male	23 (60%)	13 (46.4%)	10 (35.71%)		
Female	33 (40%)	15 (53.6%)	18 (64.29%)		
Education (%)				1.806	0.415
School level	25 (44.64%)	10 (35.71%)	15 (53.57%)		
Higher education	31 (55.36%)	18 (64.29%)	13 (46.43%)		
Work				0.076	0.783
Yes	35 (62.5%)	17 (60.71%)	18 (64.29%)		
No	21 (37.5%)	11 (39.29%)	10 (35.71%)		
Physical labour				2.947	0.086
Extremely light	18 (32.15%)	6 (21.4%)	12 (42.9%)		
Light	38 (67.85%)	22 (78.6%)	16 (57.1%)		
Extremely light	18 (32.15%)	6 (21.4%)	12 (42.9%)		
Dominant hand				1.018	0.313
Extremely light	18 (32.15%)	6 (21.4%)	12 (42.9%)		
Light	38 (67.85%)	22 (78.6%)	16 (57.1%)		
History of smoking				0.583	0.445
Yes	8 (12.5%)	4 (14.29%)	3 (10.71%)		
No	48 (87.5%)	24 (85.71%)	25 (89.29%)		
History of drinking				1.00	0.00
Yes	6 (14.29%)	4 (14.29%)	4 (14.29%)		
No	50 (85.71%)	24 (85.71%)	24 (85.71%)		
Duration of tinnitus (years)				0.087	0.778
0.5–5 (including 5 years)	37 (66.08%)	19 (67.86%)	18 (64.29%)		
> 5	19 (33.92%)	9 (32.14%)	10 (35.71%)		
History of treatment				0.311	0.575
No	20 (35.71%)	11 (39.29%)	9 (32.14%)		
Yes	36 (64.29%)	17 (60.71%)	19 (67.86%)		
Light tinnitus (THI < 16)	13 (23.21%)	4 (14.29%)	9 (32.14%)	2.504	0.114
Mild tinnitus (18–36)	13 (23.21%)	5 (17.86%)	8 (28.57%)	0.902	0.342
Severe tinnitus (58–76)	14 (25%)	7 (25%)	7 (25%)	1.000	0.000
Catastrophic tinnitus (78–100)	6 (10.72%)	4 (14.28%)	2 (7.14%)	0.747	0.388
Tinnitus side				0.220	0.639
Left or left head	30 (53.57%)	13 (46.43%)	17 (60.74%)		
Right or right head	26 (46.43%)	15 (53.57%)	11 (39.26%)		
PTA in tinnitus side					
PTA right ear	22.04 (17.72)	18.77 (9.42)	25.3 (26.02)	1.059	0.299
PTA left ear	24.83 (21.07)	27.28 (16.81)	22.38 (15.33)	0.64	0.53
Feldmann’s curve				3.170	0.075
Convergent or overlapping	53 (94.64%)	28 (100)	25 (89.29%)		
Separated or spaced	3 (5.36%)	0 (0)	3 (10.71%)		
RI-value				0.424	0.515
Incompletely positive	12 (21.43%)	5 (17.86%)	7 (25%)		
Completely positive	44 (78.57%)	23 (82.1%)	21 (75%)		
Feldmann’s curve				3.170	0.075
Convergent or overlapping	53 (94.64%)	28 (100)	25 (89.29%)		

*SD* standard deviation; *CAABT* Cochleural alternating acoustic beam therapy; *TST* traditional sound therapy; *RI* residual inhibition; *THI* tinnitus handicap inventory; *PTA* pure-tone audiometry; School level: primary, junior, and high schools; Higher education: undergraduate or postgraduate.

### Comparison of THI and VAS

**Figure 2:**
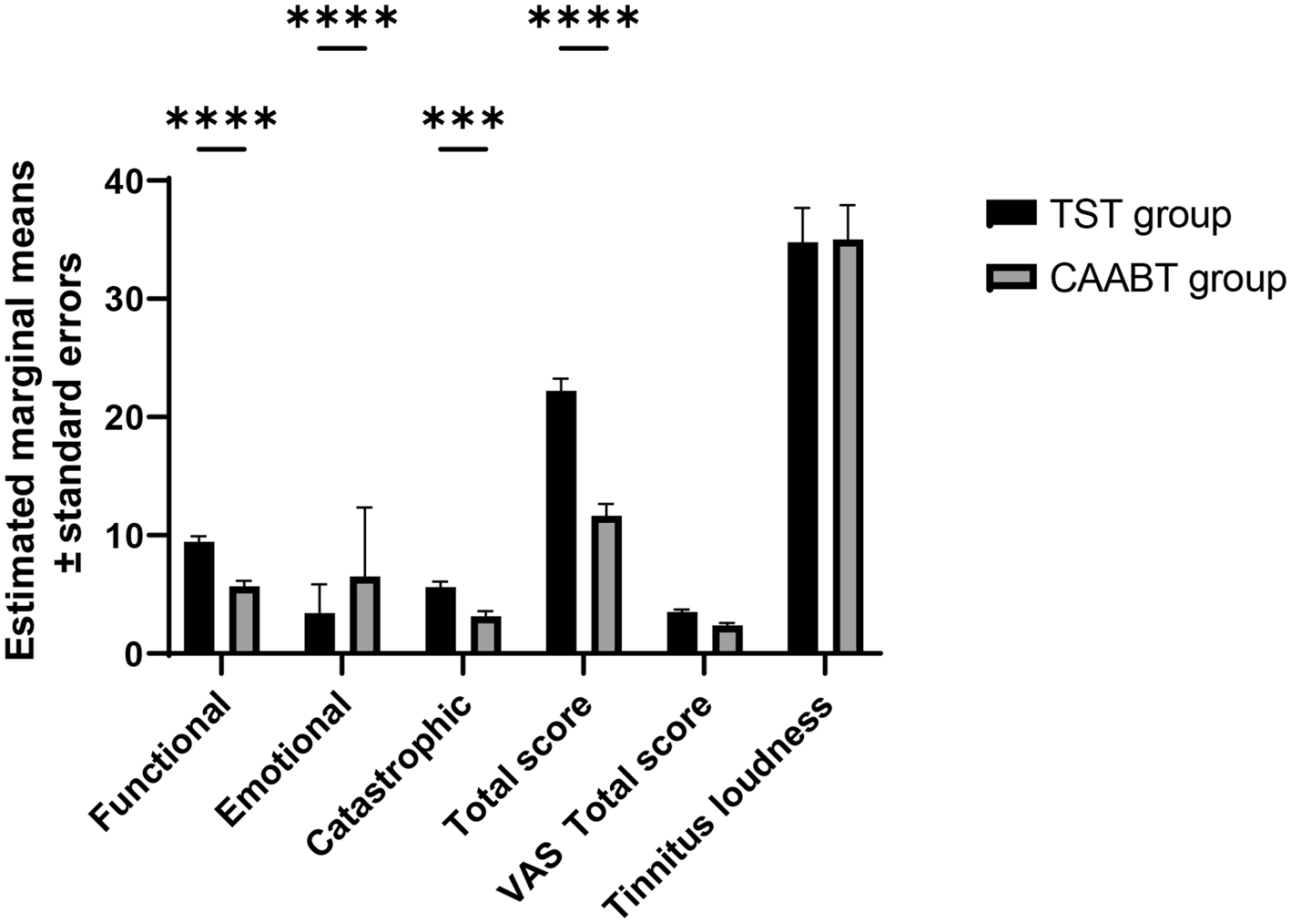
Bar chart showing the estimated marginal means ± standard errors for co-factors pre-intervention and post-intervention.

At baseline, THI and VAS ratings did not significantly differ between the two groups (*P* > 0.05). However, after three months of therapy, both groups demonstrated significant reductions in THI and VAS values after treatment (*P* < 0.05; Table 2). Furthermore, CAABT showed superiority to TST in THI Functional (*p* = 0.018), THI Emotional (*p* = 0.015), THI Catastrophic (*p* = 0.022), THI total score (*p* = 0.005) as well as VAS score (*p* = 0.022). Regarding tinnitus loudness, there were no significant differences between the CAABT group and TST group at baseline and after 3 months of therapy (*p* > 0.05; Table 2). Next, post-intervention outcomes from pre- to post-intervention were analyzed using ANCOVA with the pre-intervention measure as a covariate. The results showed that CAABT showed superiority was superior to TST in the THI functional (F = 34.165, *p* < 0.0001), THI emotional (F = 33.811, *p* < 0.0001), THI catastrophic (F = 15.161, *p* < 0.001), THI total score (F = 52.116 *p* < 0.001) and VAS score (F = 13.524, *p* < 0.001). Figure 2 demonstrates estimated marginal means and standard errors for the clinical cofactors pre-and post-intervention. We also compared the changes of variables from baseline after both interventions. The results showed that CAABT showed superiority to TST in the changes of all variables from baseline (Table 3). Significant reductions in tinnitus loudness were observed within each group after treatment (*P* < 0.01; Table 3).

**Table 2:** Outcomes of THI, VAS, and tinnitus loudness at baseline and 3 months after treatment within and between the CAABT group and TST group.

Outcome measures	CAABT group (n = 28)			TST group (n = 28)			P1	P2	*F*	*P3*
	Baseline	3 months	*P*	Baseline	3 months	*P*				
THE Functional	19.57 (11.79)	6.1 (3.31)	0.0001	16.3 (12.47)	9.1 (5.21)	0.006	0.348	0.018	34.165	< 0.0001
Emotional	15.79 (10.26)	3.4 (2.43)	0.000	11.8 (10.97)	6.5 (5.85)	0.0282	0.172	0.015	33.811	< 0.0001
Catastrophic	11.64 (5.22)	3.5 (2.96)	0.000	9.64 (4.08)	5.29 (2.68)	0.000	0.116	0.022	15.165	< 0.001
Total score	47 (25.74)	13.0 (7.02)	0.000	38 (25.51)	20.7 (11.89)	0.000	0.194	0.005	52.166	< 0.001
VAS score	5.82 (2.50)	2.5 (1.29)	0.0001	5.21 (1.81)	3.39 (1.52)	0.000	0.303	0.022	13.524	< 0.001
Tinnitus loudness	47.32 (19.97)	32.71 (23.36)	0.0187	42.3 (20.40)	32.07 (21.88)	0.0868	0.362	0.898	0.003	0.956

*CAABT* Cochleural alternating acoustic beam therapy; *TST* traditional sound therapy. THI: tinnitus handicap inventory; VAS: visual analogue scale p: comparison between before and after treatment in each group; p1, comparison between two groups before treatment; p2, comparison between two groups after treatment. P3: Comparison of estimated marginal means ± standard errors between groups.

**Table 3:** Comparison of the change from baseline.

Outcome measures	Change from baseline		
	CAABT group (n = 28)	TST group (n = 28)	*P*
THI Functional	13.2 (8.50)	7.21 (2.47)	0.0009
Emotional	12.38 (7.53)	5.5 (2.34)	< 0.0001
Catastrophic	7.89 (2.45)	4.23 (2.10)	< 0.0001
Total score	35 (10.51)	11.5 (4.67)	< 0.0001
VAS score	2.20 (0.30)	1.45 (0.45)	< 0.0001
Tinnitus loudness	15.82 (5.43)	9.81 (5.23)	0.0001

### fMRI results

**Figure 3:**
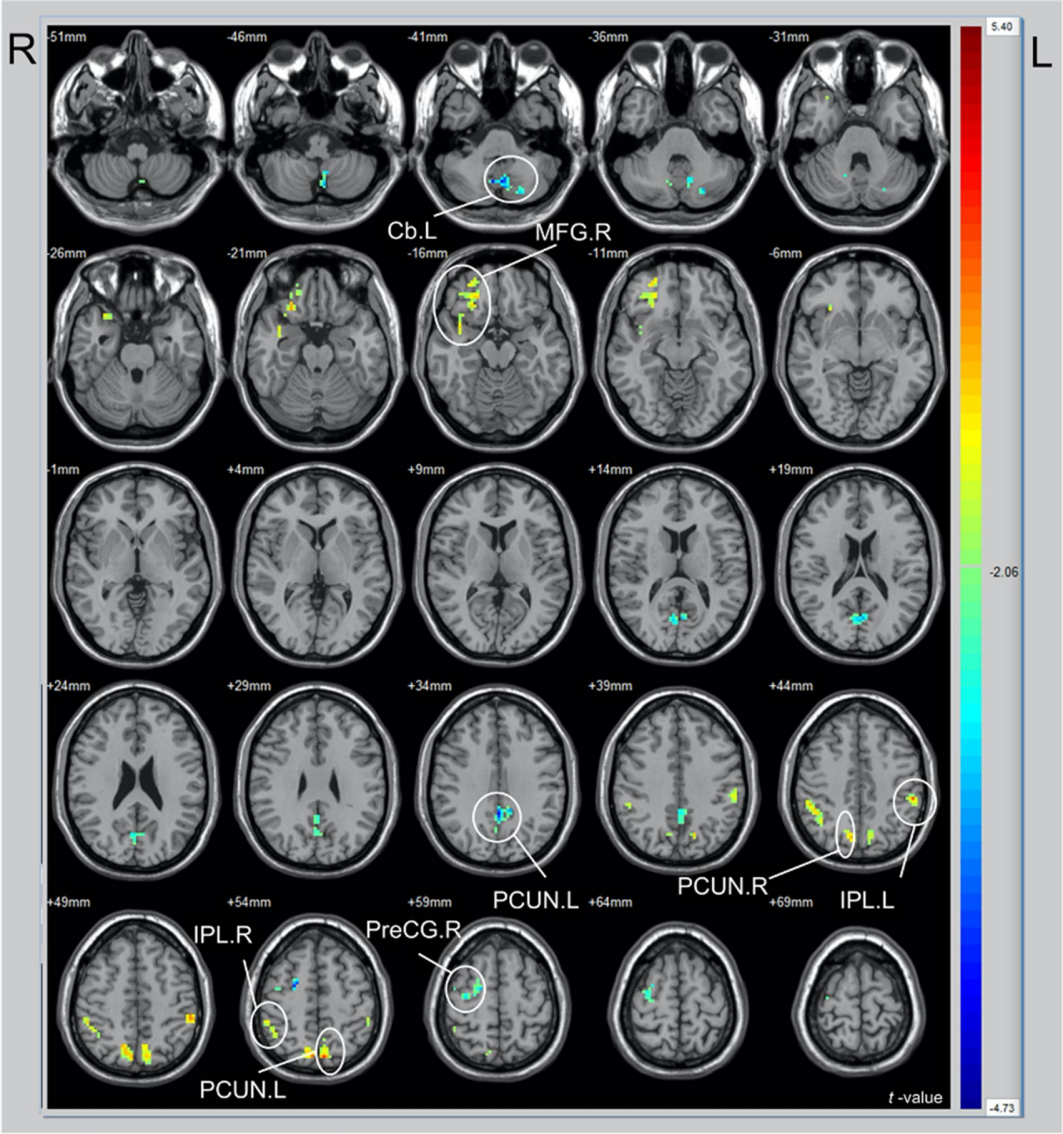
Comparison of 26 patients before and after cochleural alternating acoustic beam therapy (CAABT) (paired-samples *t*-test). *R* right hemisphere; *L* left hemisphere; *Cb* Cerebellum; *MFG* Middle frontal gyrus; *PreCG* Precentral gyrus; *PCUN* Precuneus; *IPL* Inferior parietal lobule.

**Figure 4:**
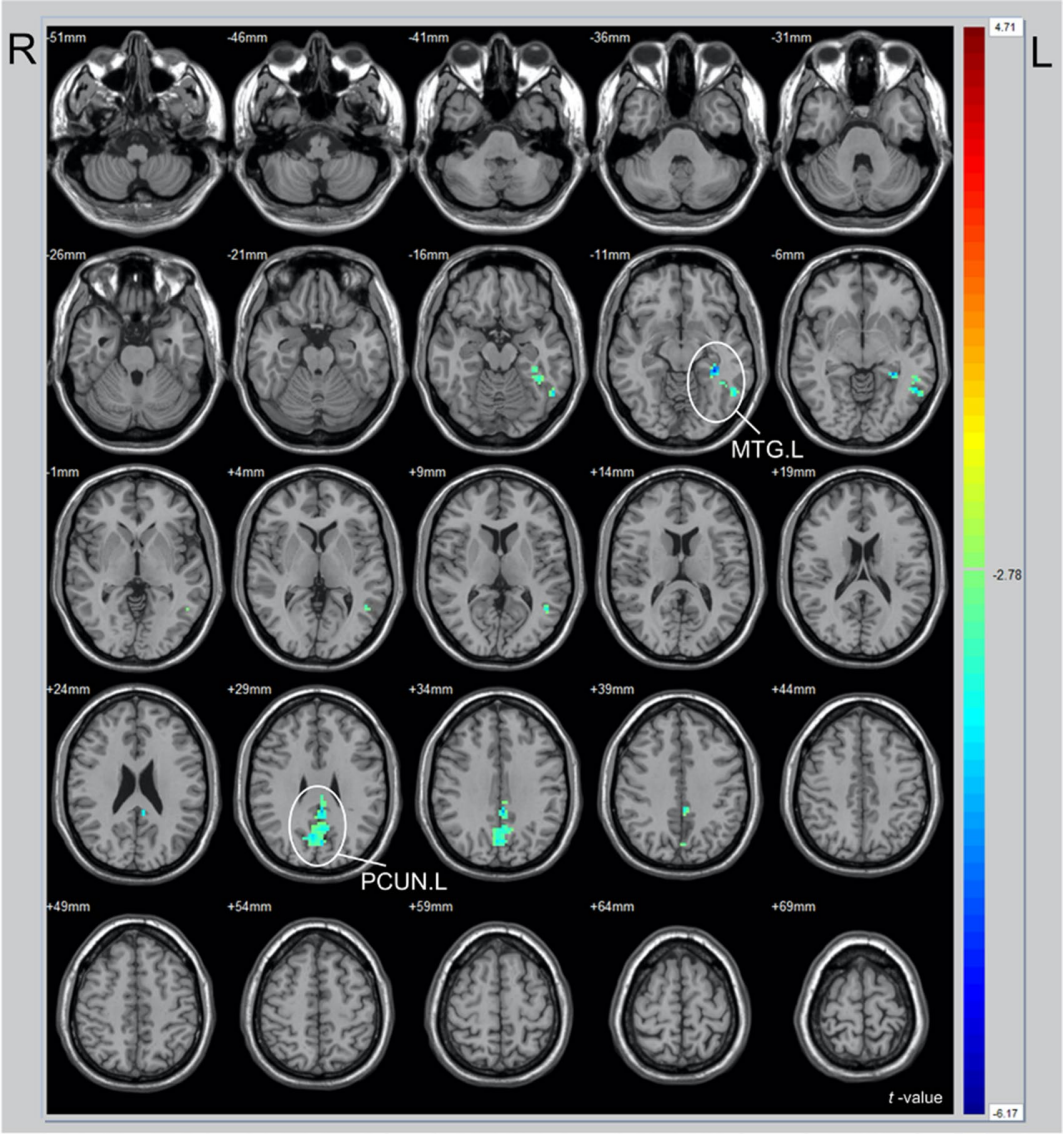
Comparison of 28 patients before and after traditional sound therapy (TST) (paired-samples *t*-test). *R* right hemisphere; *L* left hemisphere; *PCUN* Precuneus; *MTG* Middle temporal gyrus.

The CAABT group (26 participants) and TST group (27 participants) were matched with the same sex, age, and right-handedness. Paired-sample t-tests were conducted within each group to compare ReHo values before and after treatment; brain regions that exhibited significant differences were analyzed and presented on a mind map. In the CAABT group, after treatment, the ReHo values decreased in the precuneus(t = − 4.47), right precentral gyrus (PreCG, t = − 3.96)), and posterior cerebellum (t = − 3.98); the ReHo values increased in the left inferior parietal lobule (t = 3.88), right inferior parietal lobe (t = 3.29), left cuneus anterior lobe (t = 4.05), right precuneus (t = 3.86), and right middle frontal gyrus (MFG, t = 3.78). After treatment, the precuneus and left middle temporal gyrus in the TST group showed lower ReHo values (respectively t = − 4.63; t = − 6.17); no other brain areas displayed higher ReHo values. Figures 3 and 4 depict changes in various brain regions following CAABT and TST, respectively. ReHo values that are higher are displayed in red, while those that are lower are displayed in blue. The detailed information is presented in Table 4.

**Table 4:** Significance difference of signals in the same brain region.

Group	Anatomical regions	*t*	x (mm)	y (mm)	z (mm)
ReHo-CAABT	Cb.L	− 3.98	− 3	− 66	− 42
	MFG.R	3.78	27	27	− 18
	PCUN.L	− 4.47	0	− 48	36
	IPL.R	3.29	51	− 39	45
	PCUN.L	4.05	− 6	− 75	54
	PCUN.R	3.86	6	− 72	51
	IPL.L	3.88	− 54	− 33	45
	PreCG.R	− 3.96	21	− 3	57
ReHo-TMT	MTG.L	− 6.17	− 30	− 39	− 9
	PCUN.L	− 4.63	− 3	− 39	36

*R* Right hemisphere; *L* Left hemisphere; *Cb* Cerebellum; *MFG* Middle frontal gyrus; *PreCG* Precentral gyrus; *PCUN* Precuneus; *IPL* Inferior parietal lobule; *MTG* Middle temporal gyrus.

### Side effects

Participants reported no side effects throughout the treatment period.

## Discussion

Multiple studies have demonstrated the effectiveness of sound therapy for tinnitus^23–25^. These studies revealed a positive correlation between sound therapy and relief from tinnitus symptoms^26^. Furthermore, customized sound therapies effectively improve tinnitus loudness^24^. In this prospective randomized controlled trial, the effectiveness of CAABT in treating individuals with chronic idiopathic tinnitus was compared to TST in terms of tinnitus severity reduction and quality of life enhancement. After 3 months of treatment, CAABT showed superiority to TST in THI Functional (*p* = 0.018), THI Emotional (*p* = 0.015), THI Catastrophic (*p* = 0.022), THI total score (*p* = 0.005) as well as VASscore (*p* = 0.022). More interesting, CAABT showed superiority to TST in the changes of THI scores, and VAS scores from baseline. Additionally, no side effects were reported in either group. The rs fMRI results showed significant changes in the precuneus before and after treatment in both groups. Moreover, the CAABT group showed more changes in brain regions compared to the TST. Therefore, CAABT was effective in patients with tinnitus of varying severity levels.

CAABT and TST demonstrated considerable safety and effectiveness, such that objective imaging changes were observed within 12 weeks; these changes reflect the impact of sound therapy on brain function. Notably, CAABT produced significant changes from baseline in THI and VAS scores compared with TST. The significant changes observed with CAABT may be linked to its unique mechanism of inducing neuroplastic changes and desensitization to tinnitus, as opposed to the mechanisms involved in TST. The effectiveness of each approach can be influenced by the individual characteristics of the patients, the duration of therapy, and the specific components of the sound stimuli used in each protocol.

The treatment groups exhibited changes in the precuneus, emphasizing its close association with persistent tinnitus perception and centralization, considering its role as a vital component of the default mode network^27^. We found that the ReHo value of the precuneus was altered. This variability could be attributed to factors such as tinnitus duration and severity, as well as the diverse characteristics of tinnitus sounds; the small sample size might have contributed to the contradictory results. The precuneus is a functionally complex region with distinct subdivisions; the characteristics and connectivity of each subdivision and their associations with relevant networks can substantially vary.

Additionally, significant changes were observed in the left middle temporal gyrus after TST. Han et al.^28,29^ demonstrated that patients with primary tinnitus exhibited higher fractional amplitude of low-frequency fluctuations and ReHo values in the middle temporal gyrus, compared with healthy controls. Moreover, ReHo values have shown positive correlations with THI scores. Wang et al.^30^ demonstrated that the middle temporal gyrus displays abnormal function in severe tinnitus patients. We observed a decrease in the ReHo value of the middle temporal gyrus after TST (t = − 6.17), indicating reduced local neural activity and confirming treatment efficacy. Notably, the tinnitus patients in our study had normal hearing, which is consistent with Anthony's initial study on unilateral tinnitus with hearing loss, where a decrease in the ReHo value of the middle temporal gyrus was observed^31^.

Compared with the TST group, the CAABT group exhibited functional changes in more brain areas after treatment. In addition to the precuneus, seven other brain regions showed significant alterations. Although the PreCG was suspected to contribute to somatosensory and primary motor functions, recent research has revealed its involvement in auditory-related functions and speech processing. Feng et al.^32^ observed enhanced functional connectivity between the PreCG and cerebellum in tinnitus patients, leading to significant functional changes. Additionally, Wei et al.^33^ found that sound therapy can induce morphological changes in cortical thickness within the PreCG. Consistent with previous studies, we observed functional changes in the PreCG after CAABT. Notably, the PreCG is a component of the attention network; chronic tinnitus affects both the auditory and attention networks. This study revealed a decrease in the ReHo value of the PreCG after CAABT (t = − 3.96), indicating a reduction in PreCG neural activity and the alleviation of abnormality. This reduction in attention towards tinnitus may lead to better focus on non-auditory events, thereby diminishing the perception of tinnitus sounds. Furthermore, the MFG and inferior parietal lobule are critical components of the attention network. Previous studies have demonstrated that subjective tinnitus patients exhibit decreased ReHo values in the right MFG and left anterior cerebellum^28^. Additionally, fMRI studies have revealed that neurofeedback treatment can increase the ReHo value of the right MFG in tinnitus patients, which is negatively correlated with THI and VAS scores; this finding indicates that neurofeedback treatment training effectively reduces tinnitus-related pain and sound perception^34^. The inferior parietal lobule is involved in various functions, including sensory regulation, cognitive processing, and language functions; it also regulates the activity of the superior temporal gyrus, which plays a key role in tinnitus^35^. Our study revealed that the ReHo values of the right MFG and left and right inferior parietal lobule increased after CAABT(respectively MFG t = 3.78; right inferior parietal lobule = 3.29; left inferior parietal lobule t = 3.29). This result indicates enhanced local consistency in these brain regions, suggesting that patients can shift their focus from tinnitus to other sounds, thereby reducing the sensation of tinnitus. Consequently, even if subcortical centers detect the tinnitus signal, it may not reach the level of conscious perception, leading to perceptual adaptation. This improved ability to attenuate the interference and impact of tinnitus could explain the beneficial effects of CAABT. Recent studies have demonstrated that MFG and PreCG characteristics can predict the prognosis of idiopathic tinnitus and serve as a screening tool before sound therapy^36^.

CAABT affects both the attention system and the posterior cerebellum. Although the cerebellum has been associated with motor control and coordination, there is evidence that it may be involved in auditory transmission, processing, and tinnitus-related auditory hallucinations^37–39^. In a meta-analysis of fMRI studies, Chen et al.^40^ identified increased abnormal activity in multiple brain regions, including the posterior lobe of the cerebellum, among patients with subjective tinnitus. Feng et al.^32^ proposed that chronic subjective tinnitus is related to abnormal functional coupling between the cerebellum and the PreCG, superior temporal, parahippocampal, and infraoccipital gyri. Moreover, they suggested that impaired resting-state functional connectivity between the cerebellum and the cerebral cortex plays a key role in the neuropathological aspects of tinnitus. Consistent with these findings, we demonstrated a decrease in the ReHo value of the left posterior lobe of the cerebellum after CAABT in tinnitus patients (t = − 3.98). This objective result provides evidence that CAABT effectively alleviates abnormal activity in the posterior lobe of the cerebellum. However, further research is needed to determine whether CAABT helps to reduce tinnitus-related auditory hallucinations.

Overall, these findings support our initial hypothesis that individualized and consistent CAABT, based on the interaction of sound and tinnitus, is effective in the management of tinnitus. rs-fMRI can be used to evaluate gradual habituation of perception and tolerance is achieved through assessing abnormal brain activity and changes associated with tinnitus development.

This study had several limitations. First, the 12-week trial duration was relatively short for evaluating chronic tinnitus treatment efficacy; it may not be sufficient to assess the long-term efficacy of sound therapy in chronic tinnitus management. Future studies should extend the trial duration beyond 12 months to better understand the long-term effects. Second, the daily treatment time for patients was relatively short; a longer daily sound therapy duration might enhance the potential for improving tinnitus relief^9^. There is evidence that continuous sound therapy for longer periods, such as 3 h per day for > 6 months, could yield better results^8^. Third, the trial was conducted in a single center, which may limit the generalisability and stability of the findings. Therefore, future research should involve multi-center and multi-region tinnitus treatment trials.

This study also had several strengths. First, it used a computer-generated random sequence and a double-blind approach during participant allocation and data collection, which enhanced the validity and reliability of the findings. Second, the use of the same sound therapy instrument for both experimental groups ensured intervention consistency, enabling more accurate comparisons between the groups. Third, the involvement of an independent statistician in data collection and analysis added objectivity to the study's findings. Moreover, both experimental groups received identical instructional counseling.

## Conclusion

In conclusion, CAABT effectively improved the quality of life in tinnitus patients by reducing the loudness and negative emotions associated with tinnitus and by distracting attention away from tinnitus. As a component of a comprehensive tinnitus management strategy, CAABT had superiority to THI Functional, THI Emotional, THI Catastrophic, THI total scores as well as VAS scores. More interesting, CAABT showed superiority to TST in the changes of THI scores, and VAS scores from baseline. Therefore, CAABT was effective in patients with tinnitus of varying severity levels. Our findings have the potential to provide more effective interventions for chronic tinnitus by incorporating continuous improvement and innovative utilization of acoustics.

The use of rs-fMRI offers an objective evaluation of changes in brain function after treatment, providing evidence to support physiological changes in the brain and tinnitus lesion sites because of the unique treatment provided by CAABT. This imaging approach provides a unique perspective regarding the underlying mechanisms of tinnitus development and offers insights into the possibility of long-term effects through successful habituation. Therefore, this study provided a more comprehensive understanding of the efficacy of CAABT in the treatment of tinnitus. Our study has some limitations which must be considered. Firstly, the study was done at a single hospital, limiting the generalizability of our findings to other medical institutions in various settings. Secondly, our study did not take into consideration the possibility of cognitive disorders in patients with increasing age (especially 60 years old and above).

We have some suggestions on how retention in future studies. It should explore the intricate relationship between tinnitus and hearing loss, employing thorough audiometric evaluations to gauge the extent and frequency range of hearing impairment in patients. Additionally, exploring the cognitive status of individuals with tinnitus through standardized assessments can shed light on potential links between tinnitus and cognitive function. Comparative investigations into the effectiveness of diverse sound therapy techniques, including Tailor Made-Notched Music Training (TMNMT), should be pursued, evaluating their impact on tinnitus severity, quality of life, and associated neural changes using advanced neuroimaging methods. Long-term follow-up studies with larger sample sizes are warranted to ascertain the sustainability and generalizability of treatment outcomes.

### Supplementary Information

Supplementary Information.

## Data Availability

The datasets used and/or analyzed during the current study are available from the corresponding author upon reasonable request.
