# Supplementary material for: Therapy effect of cochleural alternating acoustic beam therapy versus traditional sound therapy for managing chronic idiopathic tinnitus patients
Source: Sci Rep. 2024 Mar 11;14:5900. doi: 10.1038/s41598-024-55866-0 (PMC10928112; doi:10.1038/s41598-024-55866-0)
Supplement: Supplementary file 1 — Supplementary Information. [file 41598_2024_55866_MOESM1_ESM.docx]

Supplementary Figure 1 Phonetic diagram of cochleural alternating acoustic beam therapy (CAABT).

TF: tinnitus frequency; D1, D2, D3, and D4 were intervals between stimulus sounds as with durations of 100 milliseconds, 100 milliseconds, 500 milliseconds, and 500 milliseconds, respectively. ST （the green, red, and blue lines ）represents simultaneous sound stimulation of F1,TF and F2.. F1: TF(1-10%)Hz, F2: TF(1 + 10%)Hz.


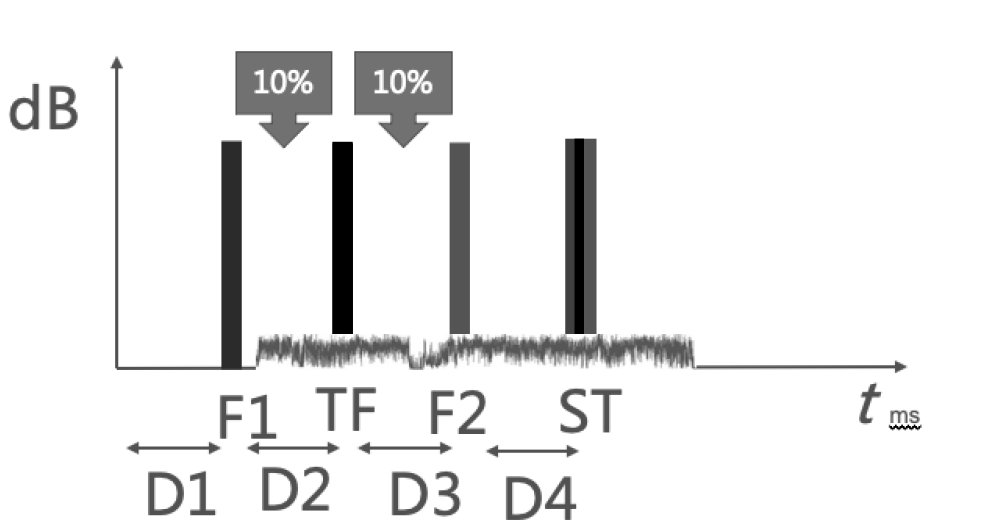


Supplementary Figure 2 Volume diagram of stimulus and background sounds in CAABT

L1 represent Minimum Masking Level（MML）, L2 represent stimulus sounds，L3 represent background sounds. L2= L1(MML)- 5dB或L3+ 5dB，L3= L1 (MML)- 10dB


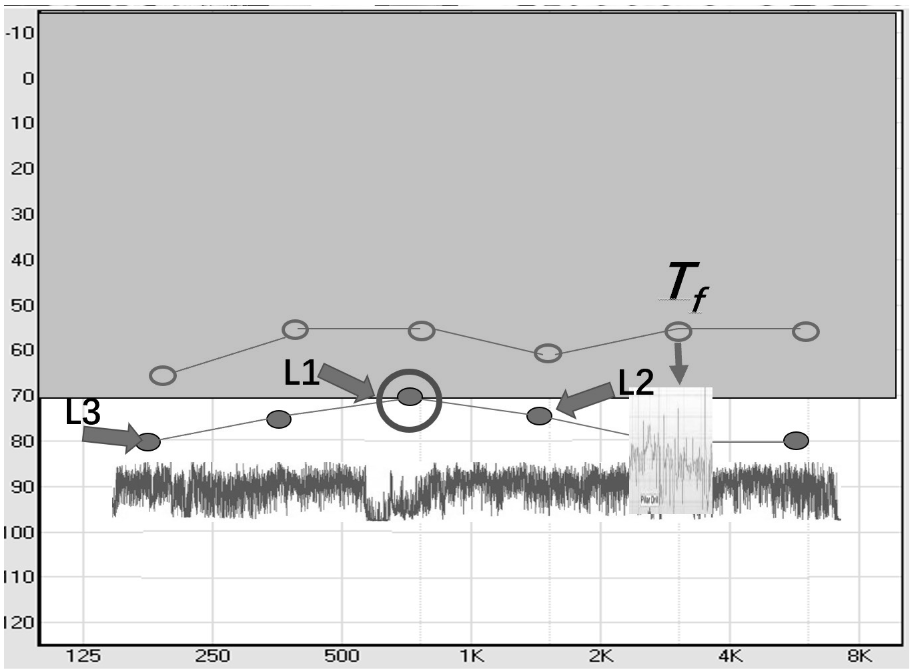


Supplementary table 1. Comparison of Hearing thresholds of each frequency

|  | Case | Mean | SD | *P* |
| --- | --- | --- | --- | --- |
| CAABT | 30 | 18.17 | 1.925 | 0.407 |
| TST | 30 | 15.83 | 2.021 |  |
| CAABT | 30 | 18.83 | 2.099 | 0.919 |
| TST | 30 | 19.17 | 2.481 |  |
| CAABT | 30 | 20.17 | 2.439 | 0.696 |
| TST | 30 | 18.67 | 2.935 |  |
| CAABT | 30 | 30.50 | 3.273 | 0.606 |
| TST | 30 | 27.83 | 3.973 |  |
| CAABT | 30 | 35.50 | 3.709 | 0.487 |
| TST | 30 | 31.50 | 4.349 |  |

CAABT: Cochleural alternating acoustic beam therapy; TST: traditional sound therapy;
